# Supplementary material for: Assessing the effectiveness of the community participation approaches to improve access to mass drug administration for trachoma elimination in a pastoral conflict area of Baringo County, Kenya
Source: PLoS Negl Trop Dis. 2025 Aug 11;19(8):e0013408. doi: 10.1371/journal.pntd.0013408 (PMC12360648; doi:10.1371/journal.pntd.0013408)
Supplement: S1 Data Tool — (DOCX) [file pntd.0013408.s001.docx]

**Appendices: Data Collection Tools**

**Appendix 1.1: Interview schedules for heads of households (male or female)**

Date:_____________________

ID:__________________________

Sub-County:______________________

Ward:______________________________________

Household no:________________________________

| Instructions:   - This form should be used for interviews to Household heads. - If the participant refuses to answer a question, circle the number of the question and do not mark any answers for that question. - After obtaining informed consent, read the following instructions to the participant: |
| --- |
| **“I am going to ask you questions about the trachoma control program, whether you have been treated by the programme, your willingness to participate by taking the drugs and preferences for being reached during treatment. Some questions about your household members focusing on the control program will be asked to you. Please answer the questions as honestly as you can remember. Your information will be kept private and this form will not have your name anywhere, you will be identified by a number only. If you have any questions or do not understand what I am asking you at any time, please ask for clarification. Some questions may prove embarrassing to you.**  **Please remember that you do not have to answer any questions that you do not want to answer and you may discontinue the interview at any time. Do you have any questions before we begin?”** |

**Socio-Demographic** **Characteristics**

1. Sex (Tick) Male ( 1) Female ( 2)

2. Age in Years_______________________

3. Marital Status (Tick)

Single (1)

Currently Married (2)

Divorced/Widowed (3)

4. Religion (Tick)

Christian (1)

Islam (2)

Non-practicing (3)

Others, specify (4) __________________

**Socio-economic characteristics**

5. Level of Education (Tick)

Never attended school (1)

Did not complete primary school (2)

Completed primary school but did not complete secondary school (3)

Completed secondary school (4)

Further studies after secondary school (5)

Others, specify (6)_____________________

6. Main occupation (Tick)

Small business (kiosk, kibanda, motorcycle operator ) (1)

Big business (shop) (2)

Housewife (3)

Salaried worker (teacher, police, chief) (4)

Pastoralist (5)

Farmer (6)

Casual laborer (7)

Others, specify (8)_____________________

7. Observe for presence of toilet facility in the home (Tick) Yes (1) No (2)

7a, If present in 7, state type

Flush (1)

Traditional pit latrine (2)

Ventilation Improved Pit (VIP) latrine (3)

No facility, bush, field (4)

Others, specify (5) ___________________

b, Share toilet with other households (Tick) Yes ( 1 ) No ( 2 )

8. Observe the type of roofing material of the main house (Tick)

Grass thatch, *makuti (1)*

Tin cans (2)

Corrugated iron sheet (3)

Brick/gall sheet (4)

Concrete (5)

Tiles (6)

Others, specify (7)_______________

9. Observe the type of main flooring material (Tick)

Earth, mud, dung, sand (1)

Wood planks (2)

Palm, bamboo (3)

Polished wood (4)

Ceramic tiles (5)

Cement (6)

Carpet (7)

Others, specify (8)________________

10, Observe the type of cooking fuel (Tick all that apply)

Electricity (1)

Gas (2)

Kerosene (3)

Charcoal (4)

Firewood straw (5)

Dung (6)

Others, specify (7)_________________

11. Sources of drinking water (Tick all that apply)

Piped into dwelling (1)

Piped into compound/plot (2)

Public tap (3)

Open well in compound/plot (4)

Covered public well (5)

Spring (6)

River, stream (7)

Pond, lake (8)

Dam (9)

Rainwater (10)

Bottled water (11)

Others, specify (12) ____________________

12. Time to water source (Tick)

Less than 15 minutes (1)

More than 15 minutes (2)

13. Water availability (Tick)

Usually available (1)

Several hours per day (2)

Once or twice per week (3)

Infrequent (4)

14. Household owns structure (Tick)

Owns (1)

Pays rent, lease (2)

No rent, with consent of owners (3)

No rent, squatting (4)

Others, specify (5) ________________________

15. Household owns land on which structure sits (Tick)

Owns (1)

Pays rent, lease (2)

No rent, with owners’ consent (3)

No rent, squatting (4)

Others, specify (5) _________________________

16. Observe for state of repair of dwelling (Tick)

Completely dilapidated shack (1)

Needs major repairs (2)

Being repaired (3)

Under construction (4)

Others, specify (6) _______________________

17. Observe how household disposes of kitchen waste and trash (Tick)

Regular collection by government (1)

Infrequent collection by government (2)

Pays for private collection (3)

Composted (4)

Dumps, buries, burns in compound (5)

Dumps in street empty plot (6)

Others, specify (7) __________________________

18. Observe for possession of durable consumer goods (Tick)

Radio (1)

Television (2)

Refrigerator (3)

Bicycle (4)

Motorcycle (5)

Car/truck (6)

Solar power (7)

**Questions on knowledge about Trachoma**

19. Do you know of anyone in your community who has trachoma?) (Tick)

1. Yes (1)
2. No (2)

20. How many such people do you know of _______________(Record a number)

21 What do you think are the causes of trachoma? (Tick)

1. Witchcraft (1)
2. Rain (2)
3. Blood (3)
4. Flies (4)
5. Others, specify (5)___________
6. Do not know/ no idea (6)

22. Do you consider yourself to be at risk of getting trachoma? (Tick)

1. Yes (1)
2. No (2)
3. Do not know (3)

**Questions on knowledge about MDA**

23. Have you heard about Mass Drug Administration for Elimination of trachoma? (Tick)

1. Yes (1)
2. No (2)

If yes in Q23, answer Q 23 a- Q23 f, If no in Q 23, go to Q 24

23a, How did you learn about the MDA in 2023?

1. Community meetings/ *Barazas* (1)
2. Town criers (2)
3. Religious institutions (3)
4. Schools (4)
5. Street banners (5)
6. Health worker (6)
7. Others, specify (7)

23b, What information did you get about the MDA in 2023?

23c, How frequently did you receive this information during the last MDA (2023)?

23d, What is your opinion of this source of information during the 2023 MDA

23e, What information were you given about the treatment regimen during the 2023 MDA?

1. About eligibility for treatment
2. About the potential side effects

23f. How would you want awareness creation to be conducted during the next round?

1. About materials/strategies_________________________________________________
2. About duration___________________________________________________________
3. About the persons creating the awareness_____________________________________

**Questions on Drug use**

24. Did you take drugs during the last MDA (2023)? (Tick)

1. Yes (1)
2. No (2)
3. Cannot remember (3)

25a. How many times have you taken trachoma drugs during MDA in the past 5 years? ______________

25b. If you have never taken trachoma drugs during MDA, give your reasons for not taking.(Tick)

1. Have been in the move in search of pasture for my animals (seasonal migration) (1)
2. Have been away (family visits, business) (2)
3. Have been away (domestic chores, grazing animals) (3)
4. Insecurity issues either conflicts and/or wild animals (4)
5. Geographical terrain (time spent due to vastness of the area) (5)
6. Others, specify ______________(6)

**Questions on perceptions of the treatment**

26. Do you consider this treatment as necessary for you? (Tick)

1. Yes (1)
2. No (2)

27. Do you have trouble swallowing the drugs? (Tick)

1. Yes (1)
2. No (2)

28. Do you have a problem with the size of the drugs given to you and/or your children? (Tick)

1. Yes
2. No

28b. If yes, specify the problem _______________________________________________________

28 c. Do you have a problem with the number of the drugs given to you and/or your children? (Tick)

1. Yes
2. No

28d. If yes, specify the problem ______________________________________________________

28e. Do you have a problem with the taste of the drugs given to you and/or your children? (Tick)

1. Yes
2. No

28f. If yes, specify the problem ________________________________________________________

29. Do you plan to take these drugs next time they are administered? (Tick)

1. Yes (1)
2. No (2)
3. Do not know (3)

30. If no, why not? (Tick as many as applicable)

- - I will be away during the distribution time (2)
  - Do not like modern medicine (3)
  - Is not necessary for me (4)
  - Fear of reactions/ complications (5)
  - Have another illness (6)
  - Insecurity in the area (7)
  - Others, specify (8)__________________________

31. Next time the drugs are administered, would you want them to be distributed the same way as this last time? (Tick)

- - 1. Yes (1)
    2. No (2)
    3. Do not know (3)

32. If no, why not? (Tick)

- Had to wait for CHW for long hours (1)
- CHW did not explain well the need to take the drug and its side effects (2)
- CHW did not have enough drugs to give (3)
- Poor interaction with the CHW (4)
- Others, specify (5) _____________________

33. How would you prefer the next MDA to be conducted?

Distribution method to reach all populations:

Distributors:

Distribution duration:

Timing of the distribution

**Do you have anything else to say?**

**THANK YOU VERY MUCH FOR YOUR TIME**
