# Supplementary material for: Assessing the effectiveness of the community participation approaches to improve access to mass drug administration for trachoma elimination in a pastoral conflict area of Baringo County, Kenya
Source: PLoS Negl Trop Dis. 2025 Aug 11;19(8):e0013408. doi: 10.1371/journal.pntd.0013408 (PMC12360648; doi:10.1371/journal.pntd.0013408)
Supplement: Attachment — Submitted filename: Observaciones manuscrito Plos (1).docx [file pntd.0013408.s002.docx]

Manuscript review**:**

**Assessing the effectiveness of the community participation approaches to improve access to mass drug administration for trachoma elimination in a pastoral conflict area of Baringo County, Kenya**

First of all, thank you very much for the opportunity to review this interesting manuscript resulting from a research study. The article evaluates the impact of community participation in improving access to mass drug administration (MDA) in the fight against trachoma in Baringo, Kenya. Using a pre-post intervention design, the study's results show a notable increase in MDA coverage, from 72.4% to 92.9%, suggesting the effectiveness of the implemented strategies. Additionally, there was an improvement in the community's knowledge about trachoma and MDA, indicating that education plays a key role. Overall, the study supports the usefulness of participatory approaches to enhance treatment coverage and recommends their expansion to advance the elimination of trachoma in the region.

Next, I respectfully wish to make a few observations that I believe could be helpful to the authors in improving the quality of the manuscript.

**In the author’s abstract**

1. Lines 22 and 23: It is suggested to review the text, as it states that the SAFE strategy was developed as a goal to eliminate trachoma. It is considered that the SAFE strategy is a "means," not a "goal."
2. Line 25: The threshold defined by the WHO for mass antibiotic administration is ≥5%, not 10% as stated in the text.

**Introduction**

3. Lines 90 to 91: In the introduction, it is stated that trachoma is the leading cause of blindness worldwide, which is incorrect because cataracts hold this position. I believe it is necessary to clarify that trachoma is the leading "infectious" cause of blindness.
4. In the introduction, I suggest describing how the distribution of medicines is planned and carried out, and how MDA coverage is calculated in Kenya. Is a population census conducted first, followed by MDA? Are the census and mass drug administration done simultaneously? Are community censuses used or those from sectors other than health, and is treatment coverage estimated through a survey? I suggest clarifying this in the introduction.

**Methodes**

5. In the methods section, participants aged 18 and older were selected for the study, but it is clear that mass administration of azithromycin should be done starting at 6 months of age. Were ethical reasons the only factor influencing the inclusion of those over 18 in the study? Does absenteeism or non-participation in previous MDA rounds in children and those under 18 explain the same reasons?
6. In the study design, I understand that the baseline coverage data is from the 2022 MDA round; is that correct? It is suggested to clarify this in the text.
7. I believe it is necessary to clarify some variables in the pre-evaluation (2022) and post-evaluation (2023) that may have significantly influenced the MDA coverage. For example: the number of people and groups distributing the medication in both rounds; the amount of time that drug distributors spent in each community conducting the MDA; whether individuals from the community (other than the health sector) participated in the first round of drug administration? If so, how was their MDA training process carried out?
8. It is suggested to clarify in the methodology what the duration of the MDA was in both pre- and post-intervention, how many health workers or drug distributors participated in the pre- and post-intervention; would the difference in number and the time spent in villages explain the increase in coverage?
9. Regarding the time between taking the medication and asking about possible adverse effects in the pre- and post-intervention phases, was the duration similar in both phases? This question considers that not all adverse events occur within the first hours.
10. Lines 165-167: Did it only take 4 days to carry out the MDA in the pre-intervention and 4 days in the post-intervention across the entire district? Please clarify the duration of the pre- and post-MDA rounds in the text.

**Results**

11. In the results, the average age of the participants is reported. Typically, the age of populations does not follow a normal distribution, and therefore, the mean and standard deviation are not the most appropriate measures of central tendency. Was any test performed to determine the age distribution of the population? What was the result? If the distribution is not normal (which would generally be expected), it is suggested to use the median and interquartile range; please describe this in the methodology.
12. Lines 137 and 138: It is mentioned, "Quantitative data collection methods were used in both the pre- and post-intervention phases." What do you mean by "quantitative methods for data collection"?
13. In Table 3, it is recommended to adjust the decimal places of the percentages, as they total 100.1%.
14. What methodology was used to calculate the impact described in Table 5 (the last column on the right)?
15. In Tables 3 and 5, are the p-values, CI, and OR in the pre-intervention, post-intervention, and impact phases adjusted or crude?
16. Line 262: In the discussion, I also suggest including a hypothesis that could explain this result: “Participants with floors made of wood/palm/bamboo also showed significantly increased access to MDA (OR = 7.46, 95% CI: 1.01-55.01, p = 0.049)”.
17. In Table 5, to facilitate the interpretation of the results, it is suggested to use the lowest-risk category as a reference, and to do so in a standardized manner for all variable categories. For example, “Never attended school” was used as the reference category, while in other categories, the reference used was the one expected to represent the lowest risk, such as “Post-secondary.” Is there any theoretical basis for this? Otherwise, it is suggested to run the models using the lowest-risk category (or the highest risk category if the focus is on highlighting protective factors), which will make the results easier to understand.
18. Tables 3 and 5 evaluate the relationship between sociodemographic factors and MDA coverage in the pre- and post-intervention phases; the reason for separating the tables and calculating the association measures (OR) separately is unclear. Does this mean that the ORs, confidence intervals, and p-values are from a bivariate analysis? It is suggested to clarify this in the table and methodology, and to perform a multivariable analysis to identify and exclude confounding variables, thus improving the study's external validity.
19. In Table 7, the following data is reported: "Proportion who affirmed to have taken trachoma drugs" (79.4) and 276 (78.9). Wouldn’t these represent the MDA coverage achieved in the pre- and post-intervention?
20. It is unclear in the post-intervention design what methodology or strategy was implemented to address the nomadic population described in the context.

**Discussion**

21. Table 3 suggests that practicing a religion other than Christianity or not practicing any religion is a protective factor or predictor of better MDA coverage, compared to Christianity. Is there any hypothesis regarding this result? It is considered appropriate to mention this in the discussion.

21. The frequency of the adverse effects described in Lines 342 to 345 is quite significant. It is considered appropriate to mention these values in comparison to those reported by other researchers, to understand whether this is something that is negatively impacting the participation of people in that district in MDA rounds.
