# Supplementary material for: Assessing the effectiveness of the community participation approaches to improve access to mass drug administration for trachoma elimination in a pastoral conflict area of Baringo County, Kenya
Source: PLoS Negl Trop Dis. 2025 Aug 11;19(8):e0013408. doi: 10.1371/journal.pntd.0013408 (PMC12360648; doi:10.1371/journal.pntd.0013408)
Supplement: Attachment — Submitted filename: Response reviewers.docx [file pntd.0013408.s004.docx]

**Comments to the Authors:**

Reviewer #1: The objective of the study is well formulated and the pre and post study design is suitable for this objective.

>> Author response: Thanks for your positive appraisal

The study population is well identified, clearly described, and the analyzed sample responds to the hypothesis or objective set for the study.

>> Author response: Thanks for your positive appraisal

The sample size evaluated for the pre- and post-intervention analysis is sufficient and representative of the study population and to infer the results; However, it is considered necessary to describe in the discussion, in the limitations, the representativeness of people under 18 years of age (who did not participate in the surveys), but who were still subject to the mass drug administration.

>> Author response: Thanks for this positive suggestion. We have added this as a limitation. See lines 966-970.

With respect to the statistical analysis, in the attached document observations are made, related to measures of central tendency used depending on the distribution of the data, the need to clarify whether the ORs, with their 95% CI and p values, are raw values or adjusted? The need to review the reason why some sociodemographic variables are separated from the model and analyzed separately is also identified

>> Author response: Thanks for this positive comment. We have clarified in the mentioned tables that the p-values, CI, and OR were unadjusted. Please see the revised Tables 4 and 6. Table 4 reports selected socio-demographic factors, while Table 6 reports selected socio-economic factors.

To facilitate the understanding of the article, the authors are suggested to use the lowest risk category as the reference category for the multivariate models, or at least standardize the lowest or highest risk category in all analyses.

>> Author response: Thanks for this positive comment. We have used the lowest risk category for all the factors and revised the text appropriately. Please see Tables 4 and 6, lines 313-345, lines 425-458, and lines 72-81.

It is considered convenient to describe in the methodology the profile of the people who delivered the MDA and the training process they underwent, especially because a high frequency of adverse reactions to medications is reported.

Author response: Thanks for this positive comment. The treatment during MDA was given by trained community drug distributors. We appreciate the reviewer's suggestion to describe the profile of the people who delivered the MDA and their training process. It's important to clarify that the adverse reactions data reported in our study reflect the information participants recalled being given to them by drug distributors regarding treatment *potential* side effects, not directly observed adverse events, see lines 791-801.

It is considered necessary that the methodology describe how the mass administration of medications is carried out, the recording of data and the assessment of the coverage achieved.

>> Author response: Thanks for this comment. We have added some background information on MDA in the Introduction section, lines 123-134.

It is considered appropriate to make explicit the strategy used in the intervention to ensure that nomadic people receive treatment.

>> Author response: We thank the reviewer for highlighting the need for clarity regarding the implemented strategies to address the nomadic population needs in the intervention phase. As part of our community participatory approach, our **"Effective Communication and Community Engagement"** strategy was specifically designed to include approaches tailored to reach this population. This strategy incorporated **engagement of nomadic community leaders** and **information dissemination through their existing community networks**, as well as **flexible timing** of persons tasked with MDA delivery to accommodate their movements. These components are elaborated in detail within the **"The tested interventions"** section of the methodology, see lines 263-290.

Reviewer #2: I'm unclear on the timing of the pre-intervention, intervention, and post-intervention periods. On line 146 you say the intervention was the November 2022 MDA, but in line 166, the pre-intervention survey was done in February 2023 and the post-intervention survey was October 2023.

>> Author response: Thank you for pointing out the lack of clarity in the timeline. To clarify:

- The **pre-intervention survey** was conducted in February 2023, and it collected data related to the **2022 MDA campaign** (which, as stated in line 176, occurred in November 2021).
- The **intervention** (the community participation strategies) was implemented in May 2023, *concurrently* with the **2023 MDA campaign**.
- The **post-intervention survey** was conducted in October 2023, collecting data related to the **2023 MDA campaign**.

We acknowledge that the original wording was ambiguous, and we have revised line 184 in the manuscript to explicitly state this timeline.

Please include a brief summary of the MDA history of the chosen area. (To help contextualize some of the results about the number of consecutive MDAs people reported taking.)

>> Author response: Thank you for this question. We have added a new subsection, “MDA history of the study area”, in lines 237-262 to address this question.

Were pre-intervention survey results used to formulate the tested interventions, or were these chosen a priori?
>> Author response: Thank you for this question. To confirm, the pre-intervention survey results were indeed used to inform the formulation of the tested interventions. Specifically, the findings from the pre-intervention survey in the following areas directly shaped the design of our interventions:

- Participants' suggested strategies for improving MDA access.
- Participants' perceptions of the treatment and drug distributors.
- Participants’ information regarding eligibility and potential side effects.
- Participants reported sources of information about MDA.

These results provided crucial insights that allowed us to identify key areas for improvement and to tailor our interventions to the specific needs and challenges of the study population. We targeted these areas to enhance community engagement and ultimately improve MDA coverage.

More information about the intervention would be useful in order to contextualize some of the results, especially around the suggested strategies on how MDA should be conducted. For example, participants indicated a preference for early morning distribution, but we don't know the timing of the MDA that was actually done.

>> Author response: Thank you for this question. Please note that the actual MDA was done by the Ministry of Health; we have added some details in a new subsection, “MDA history of the study area”, in lines 237-262. The MDA timing was not more about early morning distribution but rather a better planned implementation that does not keep people waiting for too long at the places where they are.

Why were participant responses pooled, and how was this done?
>> Author response: Pooling of responses was done specifically for the open-ended questions. For example, we pooled responses from a question like information received during MDA and categorized the responses into trachoma elimination, dosage, importance of taking drugs, treatment eligibility etc, see line 228.

If possible, consider including the quantitative survey instrument as a supplementary file.
>> Author response: This is well noted. We have added the quantitative survey instrument as supplementary material.

Did the information about toilet coverage come solely from the respondents' interviews or were these directly observed?
>> Author response: The presence of a toilet facility was directly observed by the interviewer during the household survey. However, the interviewer was allowed to probe in case he/she was unsure of the presence and type of toilet facility around the homestead.

I don't see a definition of MDA access. Is this if people were offered the drug, swallowed the drug, or something else? Please clarify.

>> Author response: We have now provided a definition of MDA access, please see lines 930-931.

The analysis plan was not reviewed for review; observations on this topic are made based on the described methodology. In general, the data is well presented, however, recommendations and questions are made to the authors about some aspects and data written in the tables that could be improved. It is considered necessary to adjust some tables in which the risk/protective factors are reported, so as to explain whether the OR, CI and p values are adjusted or crude.

>> Author response: We have clarified in the mentioned tables that the p-values, CI, and OR were unadjusted. Please see the revised Tables 4 and 6.

Reviewer #2: The age range listed in line 220 is 16-101, however in the ethics section, you only mention inclusion of respondents over the age of 18. What is the cause for the discrepancy?
>> Author response: The study planned to interview participants who were aged 18 years and above, and were household heads. However, a few household heads were aged below 18 years and were termed as mature minors. They therefore consented on their own behalf and answered questions on behalf of their family members.

I think you could add two columns to Table 3 for the number of participants in each category with access to MDA pre- and post-intervention, respectively.
>> Author response: We have added two columns to Table 4, as recommended, showing the number of participants in each category with access to MDA in the pre- and post-intervention phases, respectively.

I would switch the order of Tables 3 and 4, to present socio-demographics of respondents prior to odds ratios, but that's a personal preference.
>> Author response: Table 3 gives the socio-demographic factors first, then Table 4 gives the odds ratios for the socio-demographic factors. The same order is followed for socio-economic factors in Tables 5 and 6.
In Table 4, the pre-intervention drinking water source percents are incorrect; likely a copy paste error.
>> Author response: Thank you for pointing out the error in Table 5 (formerly Table 4). We have corrected it appropriately.

To the header of table 4, please make it clear that the numbers in brackets are percents.
>> Author response: Thank you for this suggestion. We have revised the header of Table 5 (formerly Table 4) to clarify that the values in brackets represent percentages.

In line 270, you indicate that participants were asked to identify the number of people they knew who were infected. As infection status would require analysis of ocular material via PCR, I'm wondering in instead you meant "affected"? (Affected how so, though? Experiencing trichiasis?)
>> Author response: Thank you for raising this important point about the terminology used in line 721. We acknowledge that "infected" typically implies a confirmed diagnosis, often through laboratory testing such as PCR. To clarify, when we asked participants to identify the number of people they knew who were "infected" with trachoma, we were referring to their *perceived* knowledge of individuals in their community with signs or symptoms suggestive of trachoma, as understood by the participant. This was based on their own observation and community understanding of the disease, not on any formal medical diagnosis or laboratory confirmation. We have now put the term infected in quotes.

In Table 6, I don't understand the placement of "CHWs" in the table.
>> Author response: Thank you for pointing out the potential confusion regarding the placement of "CHWs" in Table 7 (formerly Table 6). We have removed this to avoid this confusion.

Please define CHW and CHV at first use. Are these interchangeable? In Fig 2, the top panel has CHV and the bottom has CHW.

>> Author response: Thank you for pointing out the need for clarification regarding the abbreviations CHWs and CHVs. We have defined the two words at first use in the text, as follows; CHWs refers to Community Health Workers, and CHVs refers to Community Health Volunteers. These terms are not interchangeable. Regarding Figure 2, the top panel shows reported channels of communication for MDA during the pre-intervention phase, where both CHVs and CHWs were reported. The bottom panel shows reported channels of communication for MDA during the post-intervention phase, where only CHWs were reported.

Line 325: What is the translation of "wazee wa nyumba kumi", please?
>> Author response: “Wazee wa nyumba kumi” is a Swahili phrase meaning “elder of ten households” referring to a community policing strategy in Kenya where households are organized into groups of ten and each group is headed by an elder.

I don't understand the results in lines 369-370: how could a higher percentage of people have taken the drugs during the last MDA than those who had ever taken trachoma drugs?
>> Author response: The percentage of those who had taken the drugs was out of those who had ever taken the drugs, while the percentage of those who had ever taken the drugs was out of the total of participants during that phase of the study, which was 350 participants. This has been clarified in the text. Please see lines 832-833.

Line 380: Is this "significant" in a statistical sense, or just large? Significantly higher than pre-intervention?

>> Author response: Thank you for seeking clarification. We confirm that the increase was statistically significant. The proportion of participants who reported taking trachoma drugs during the last MDA increased from 165 (72.4%) pre-intervention to 249 (92.9%) post-intervention. This difference was statistically significant (Difference = -0.205, z = -5.68, p < 0.001), as is already stated in lines 842-843 of the revised manuscript.

**Conclusions**

Reviewer #1: A limitation of the study is adequately described, based on the absence of a control group, but it is suggested to review the relevance of including other limitations associated with the non-participation of children under 18 years of age, who did not participate in the study, but did participate in the mass drug administration. The topic of study is relevant; Understanding the reasons why coverage goals are not achieved in mass drug administration is essential for trachoma elimination programs, as is evaluating the contribution of interventions to improve coverage.

>> Author response: Non-participation of children under 18 years of age was added as a limitation. See lines 967-971.

Reviewer #2: I'm not sure if the conclusions that effective communication and enhanced capacity building of MDA teams influenced access to MDA (line 450) is supported by the data presented. (Maybe this is presented and the connection was just not obvious to me! In which case, please clarify.) In general, the results presented allow you to make conclusions on how socio-economic factors are associated with access to MDA pre- and post-intervention. It's less clear that you are able to make conclusions about the effectiveness of the different tested interventions, as ideally, you'd have a control group who did not receive the tested interventions. (And you do mention this in the limitations.)

>> Author response: We thank the reviewer for highlighting this important point regarding the support for our conclusions on the influence of effective communication and enhanced capacity building on access to MDA. We acknowledge that without a direct control group, establishing definitive causality is challenging. However, our analysis of the baseline survey data revealed specific issues reported by participants that directly informed the design of our intervention strategy. Specifically, several participants cited **poor interaction with drug distributors (CHWs)**, **unclear explanations from CHWs regarding the importance of taking trachoma drugs**, and instances of **CHWs not having sufficient drugs to distribute**. These findings directly informed our decision to invest in enhanced communication strategies and capacity building for the MDA teams, including CHWs. Following the implementation of these strategies, we observed a significant improvement in MDA access (as presented in lines 840-848). While we cannot definitively attribute this improvement solely to these interventions due to the lack of a control group, the temporal association, coupled with the fact that the interventions directly addressed the identified barriers from the baseline data, strongly suggests a positive influence. As the reviewer correctly notes, the absence of a control group is a limitation that we have acknowledged in our manuscript.

In line 470, you state that the number of participants aware of MDA increased, but from the data this was not significant. I think the conclusion should be qualified with this information.

>> Author response: Thank you for your comment. Even though the increase in MDA awareness was not significant, it contributed to the overall access to MDA in the study area, as stated in the conclusion section.

From Fig. 2, aren't most people informed of MDA through Chief's meetings (pre-intervention) and Market place (post-intervention)? I'm not clear on why the conclusion is then that most people are informed by CHWs. (line 472).
>> Author response: Thank you for raising this point regarding CHWs as the primary source of MDA information. We acknowledge that Figure 2 shows Chief's meetings as a prominent source (pre-intervention) and the marketplace (post-intervention). However, our conclusion that CHWs are the primary source is based on the *overall* frequency of information sources across both pre- and post-intervention periods. When the frequencies for each source are combined across both time points, CHWs have the highest total frequency compared to other sources. We have slightly revised the conclusion appropriately.

In my opinion, the high number of respondents reporting a negative interaction with a CHW also provides evidence that CHW capacity building and training is paramount.
>> Author response: We agree with the reviewer that the high number of respondents reporting negative interactions with CHWs underscores the paramount importance of CHW capacity building and training. As suggested, we have strengthened the connection between the finding regarding "poor interactions by CHWs with participants" and the need to invest in CHW training within the discussion section of the revised manuscript (see lines 943-952).

Please include in the limitations an acknowledgement of biases associated with survey-taking, such as social desirability bias (i.e. were respondents biased toward giving answers they perceived as being "correct"?). Was anything done to address these biases, and how do you think they may have affected the results?

>> Author response: We have added the social desirability bias as a limitation, see lines 971-976.

**Editorial and Data Presentation Modifications?**

Reviewer #1: It is recommended that tables have footer explanations, to understand whether the ORs are crude or adjusted.
>> Author response: This has been addressed. It is now clearly indicated on the tables that the odds ratios are unadjusted. Please see Tables 4 and 6.
It is suggested that the authors standardize the reference category for the OR analyses; In some variables it is the lowest risk category and in others the highest risk.

>> Author response: We have corrected this and used the lowest risk category in all the variables. Please see Tables 4 and 6.

Reviewer #2: Data presentation modification suggestions have been included in the relevant sections above.

>> Author response: Thanks for this positive appraisal.

**Summary and General Comments**

Reviewer #1: Manuscript review:
Assessing the effectiveness of the community participation approaches to improve access to mass drug administration for trachoma elimination in a pastoral conflict area of Baringo County, Kenya
First of all, thank you very much for the opportunity to review this interesting manuscript resulting from a research study. The article evaluates the impact of community participation in improving access to mass drug administration (MDA) in the fight against trachoma in Baringo, Kenya. Using a pre-post intervention design, the study's results show a notable increase in MDA coverage, from 72.4% to 92.9%, suggesting the effectiveness of the implemented strategies. Additionally, there was an improvement in the community's knowledge about trachoma and MDA, indicating that education plays a key role. Overall, the study supports the usefulness of participatory approaches to enhance treatment coverage and recommends their expansion to advance the elimination of trachoma in the region.
Next, I respectfully wish to make a few observations that I believe could be helpful to the authors in improving the quality of the manuscript.
In the author’s abstract
1. Lines 22 and 23: It is suggested to review the text, as it states that the SAFE strategy was developed as a goal to eliminate trachoma. It is considered that the SAFE strategy is a "means," not a "goal."
>> Author response: Thank you for this important clarification. We have reviewed the text and corrected the description of the SAFE strategy in lines 21 - 23 of the revised manuscript. The SAFE strategy is now accurately referred to as a *means* to eliminate trachoma, rather than the goal itself.

2. Line 25: The threshold defined by the WHO for mass antibiotic administration is ≥5%, not 10% as stated in the text.

>> Author response: Thank you for raising this point about the threshold for mass antibiotic administration. Current recommendations from WHO are to perform at least three annual MDAs prior to an impact survey when baseline TF1–9 prevalence is 10–29.9%, and at least five MDAs before an impact survey when baseline TF1–9 prevalence is 30-49.9%, as given in the WHO (2010) Report of the third Global Scientific Meeting on Trachoma Elimination (reference [12]), please see the following decision tree (https://www.trachoma.org/sites/default/files/documents/2022-08/decision_diagram_ENGLISH.pdf).

**Introduction**
3. Lines 90 to 91: In the introduction, it is stated that trachoma is the leading cause of blindness worldwide, which is incorrect because cataracts hold this position. I believe it is necessary to clarify that trachoma is the leading "infectious" cause of blindness.
>> Author response: Thank you for this important clarification. We have revised the text in line 111 of the revised manuscript to accurately state that trachoma remains the leading *infectious* cause of blindness globally.

4. In the introduction, I suggest describing how the distribution of medicines is planned and carried out, and how MDA coverage is calculated in Kenya. Is a population census conducted first, followed by MDA? Are the census and mass drug administration done simultaneously? Are community censuses used or those from sectors other than health, and is treatment coverage estimated through a survey? I suggest clarifying this in the introduction.
>> Author response: We appreciate the reviewer's suggestion to provide more detail on the planning and execution of mass drug administration (MDA) and the calculation of MDA coverage in Kenya. We have included in the introduction section a paragraph that details the planning and how MDA coverage is calculated. Please check lines 123-134.

**Methods**
5. In the methods section, participants aged 18 and older were selected for the study, but it is clear that mass administration of azithromycin should be done starting at 6 months of age. Were ethical reasons the only factor influencing the inclusion of those over 18 in the study? Does absenteeism or non-participation in previous MDA rounds in children and those under 18 explain the same reasons?

>> Author response: Thank you for raising this important point regarding the age selection for our study. We acknowledge that mass administration of azithromycin in MDA programs typically includes individuals starting from 6 months of age. The decision to include only participants aged 18 and older in our study was primarily influenced by the study design, and ease of consenting (ethical considerations). Specifically, we required participants to provide their own informed consent (**Informed Consent**), and this is ethically challenging and marginally expensive to obtain directly from children in this context. We recognize that this age restriction is a limitation, as it excludes a significant portion of the population that is targeted for MDA, we have added this as one of our study limitations.

6. In the study design, I understand that the baseline coverage data is from the 2022 MDA round; is that correct? It is suggested to clarify this in the text.

>> Author response: Thank you for seeking clarification on the source of the baseline data. We confirm that the pre-intervention survey, which provides the baseline data, was conducted with reference to the November 2021 MDA campaign. As stated in lines 174-177, the November 2021 MDA served as the basis of inquiry. Just to put the timelines clear, the actual data collection for pre-intervention survey was done in February 2023, MDA (intervention) was done in May 2023, and the post-intervention survey was done in October 2023.

7. I believe it is necessary to clarify some variables in the pre-evaluation (2022) and post-evaluation (2023) that may have significantly influenced the MDA coverage. For example: the number of people and groups distributing the medication in both rounds; the amount of time that drug distributors spent in each community conducting the MDA; whether individuals from the community (other than the health sector) participated in the first round of drug administration? If so, how was their MDA training process carried out?

>> Author response: We thank the reviewer for highlighting the critical need to clarify potential variables in the pre- (2021 MDA, used as the basis of inquiry) and post-evaluation (2023 MDA) that could have influenced MDA coverage. We agree that factors such as the number of drug distributors, the time spent in communities, and the involvement of community members in drug administration are important to consider. Our review of the MDA implementation processes for both the 2021 and 2023 campaigns indicates that the **number of individuals and teams involved in drug distribution was largely consistent**. Similarly, the **planned duration of the MDA campaign within each community was also intended to be consistent** between the two rounds. Furthermore, the **involvement of community members beyond the formal health sector in drug administration and their associated training processes, followed a generally similar structure** in both the 2021 and 2023 MDAs. The general consistency in these key operational aspects of the MDA campaigns helps to suggest that the observed changes in MDA coverage are more likely related to the community participation interventions we implemented prior to the 2023 MDA, rather than significant variations in the drug administration process itself.

8. It is suggested to clarify in the methodology what the duration of the MDA was in both pre- and post-intervention, how many health workers or drug distributors participated in the pre- and post-intervention; would the difference in number and the time spent in villages explain the increase in coverage?

>> Author response: We appreciate the reviewer's keen attention to detail and the important questions raised regarding the duration of the MDA campaigns and the number of participating health workers/drug distributors in the pre- and post-intervention phases. To clarify, our study was not involved in offering the actual MDA, but rather assessed how community participation can be increased to improve MDA uptake.

Our study design involved:

1. **A pre-intervention assessment:** Utilizing data from the **2021 MDA campaign** as a baseline to identify groups with consistently low participation and access. This phase also involved assessing the barriers related to contextual, social, environmental, and programmatic factors hindering their engagement with MDA.
2. **The implementation of community participation interventions:** These interventions, conducted before the 2023 MDA, focused on engaging stakeholders at the county, sub-county, and community levels. The aim was to improve community awareness about trachoma, the importance of treatment, and the upcoming MDA campaign.
3. **A post-intervention assessment:** Evaluating the impact of these interventions on access to MDA during the **2023 MDA campaign**.

Regarding the duration of the MDA and the number of health workers/drug distributors involved, these factors were reported to be **consistent** between the **2021 MDA** (our baseline reference) and the **2023 MDA** (the MDA following our interventions). Therefore, differences in the number of health workers, drug distributors, or the time spent in villages are unlikely to explain any observed changes in MDA access between these two campaigns.

Our interventions specifically targeted improvements in community engagement and awareness through enhanced communication and stakeholder involvement, rather than altering the operational aspects of the MDA delivery itself (such as the number of distributors or the campaign duration). Just to clarify, both 2021 and 2023 MDAs (and all the previous MDAs) were offered by the Ministry of Health, separate from this study. These MDAs usually follow similar processes.

9. Regarding the time between taking the medication and asking about possible adverse effects in the pre- and post-intervention phases, was the duration similar in both phases? This question considers that not all adverse events occur within the first hours.

>> Author response: We appreciate the reviewer's point regarding the time between taking the medication and asking about possible adverse effects. To clarify, our study did not directly assess participants' *experienced* adverse effects. Instead, our questions in both the pre- and post-intervention surveys focused on the **information participants recalled being given by the drug distributors regarding the *potential* side effects of the drugs**.

The pre-intervention survey (referring to the 2021 MDA) and the post-intervention survey (referring to the 2023 MDA) both included questions aimed at understanding whether the drug distributors were effectively communicating the correct information about who was eligible for treatment and what potential side effects they might expect. Therefore, the "duration" the reviewer refers to is more about the participants' recall of the information provided to them during the MDA campaigns.

The differing recall periods (longer for the 2021 MDA compared to the 2023 MDA) could still influence the accuracy and detail of the information participants remembered receiving. We have clarified this in the revised manuscript that this survey assessed the recall of information provided, rather than directly reported adverse events, and we have acknowledged the potential impact of the different recall periods on these responses.

10. Lines 165-167: Did it only take 4 days to carry out the MDA in the pre-intervention and 4 days in the post-intervention across the entire district? Please clarify the duration of the pre- and post-MDA rounds in the text.

>> Author response: We thank the reviewer for seeking clarification on the duration mentioned. The **four days** mentioned in the context of pre- and post-intervention refer to the **duration of our survey data collection periods**, not the MDA campaigns themselves. This study did not conduct MDA, the MDA was done by the Ministry of Health. We only assessed the impact of the community participatory approaches we developed on the MDA uptake and access.

Results
11. In the results, the average age of the participants is reported. Typically, the age of populations does not follow a normal distribution, and therefore, the mean and standard deviation are not the most appropriate measures of central tendency. Was any test performed to determine the age distribution of the population? What was the result? If the distribution is not normal (which would generally be expected), it is suggested to use the median and interquartile range; please describe this in the methodology.

Author response: We thank the reviewer for raising this important statistical consideration. We have made revisions and calculated the median age of our participants alongside the interquartile ranges. Please see lines 303-310, and Table 3.

12. Lines 137 and 138: It is mentioned, "Quantitative data collection methods were used in both the pre- and post-intervention phases." What do you mean by "quantitative methods for data collection"?

Author response: We thank the reviewer for pointing out the need for more detail regarding the 'quantitative data collection methods’. In both the pre- and post-intervention phases, our primary method was **household surveys using structured questionnaires**. These questionnaires, administered through face-to-face interviews by trained research assistants, included both closed and open-ended questions assessing socio-demographic factors, socio-economic factors, knowledge about trachoma and MDA, and perceptions related to the MDA drugs. This approach enabled us to collect quantitative data on key indicators relevant to our study objectives.

13. In Table 3, it is recommended to adjust the decimal places of the percentages, as they total 100.1%.

Author response: We thank the reviewer for pointing out the discrepancy in the total percentage for the age group variable in Table 4 (formerly Table 3). This was an oversight, likely due to rounding of individual percentages to one decimal place. This has now been corrected in the revised manuscript to ensure the percentages sum to exactly 100%.

14. What methodology was used to calculate the impact described in Table 5 (the last column on the right)?

Author response: The intervention impact was estimated using **generalized linear models (GLMs)**. Specifically, we included a **time difference** (representing the change from pre- to post-intervention) as an **interaction term** within these models. This interaction term allowed us to assess how the outcome variable changed over time. Please find the details on the statistical analysis section, lines 227-235.

15. In Tables 3 and 5, are the p-values, CI, and OR in the pre-intervention, post-intervention, and impact phases adjusted or crude?

Author response: Thank you for this important observation. We have clarified in the **headers** of Tables 4 and 6 that the p-values, confidence intervals (CI), and odds ratios (OR) presented for the pre-intervention, post-intervention, and impact phases are **unadjusted**.

16. Line 262: In the discussion, I also suggest including a hypothesis that could explain this result: “Participants with floors made of wood/palm/bamboo also showed significantly increased access to MDA (OR = 7.46, 95% CI: 1.01-55.01, p = 0.049)”.

Author response: Thank you for highlighting this point. We have included a hypothesis to explain this result in lines 926-927.

17. In Table 5, to facilitate the interpretation of the results, it is suggested to use the lowest-risk category as a reference, and to do so in a standardized manner for all variable categories. For example, “Never attended school” was used as the reference category, while in other categories, the reference used was the one expected to represent the lowest risk, such as “post-secondary.” Is there any theoretical basis for this? Otherwise, it is suggested to run the models using the lowest-risk category (or the highest risk category if the focus is on highlighting protective factors), which will make the results easier to understand.

Author response: Thank you for this suggestion regarding the reference categories in Tables 4 and 6. We agree that using the lowest-risk category as the reference in a standardized manner across all variables will improve the interpretability of our results. We have now corrected this in the revised Tables 4 and 6.

18. Tables 3 and 5 evaluate the relationship between sociodemographic factors and MDA coverage in the pre- and post-intervention phases; the reason for separating the tables and calculating the association measures (OR) separately is unclear. Does this mean that the ORs, confidence intervals, and p-values are from a bivariate analysis? It is suggested to clarify this in the table and methodology, and to perform a multivariable analysis to identify and exclude confounding variables, thus improving the study's external validity.

Author response: Thank you for raising this important point about the tables. Table 4 (formerly Table 3) assessed the relationship between sociodemographic factors and MDA access, while Table 6 (formerly Table 5) focused on socioeconomic factors and MDA access. The separation was to present these two sets of factors distinctly.

We confirm that the odds ratios (ORs), confidence intervals (CIs), and p-values presented in these tables are the results of **bivariate (univariable) analyses**, examining the association of each individual factor with MDA access in the pre-intervention, post-intervention, and intervention impact. We have clarified this explicitly in the table captions and within the methodology section.

We understand the reviewer's suggestion to perform a multivariable analysis to identify and control for potential confounding variables, which could strengthen the study's external validity. We did perform multivariable analysis, however, only few individual factors demonstrated statistical significance. None were significant in the impact analysis. Based on this, we only presented the univariable analysis.

19. In Table 7, the following data is reported: "Proportion who affirmed to have taken trachoma drugs" (79.4) and 276 (78.9). Wouldn’t these represent the MDA coverage achieved in the pre- and post-intervention?

Author response: Thank you for pointing out the potential misinterpretation of the data in Table 8 (formerly Table 7). The values "Proportion who affirmed to have taken trachoma drugs" (79.4) and (78.9) **do not represent the actual MDA coverage achieved in the pre- and post-intervention phases.** Instead, these figures represent the proportion of survey participants who responded affirmatively to the question: **"Do you plan to take these drugs the next time they are administered?".** We acknowledge that the current language in the table is misleading. We have modified the specific row in Table 7 to accurately reflect the question asked to, **"Proportion who affirmed intent to take trachoma drugs in future MDA."** This will prevent misinterpretations of this table’s result.

20. It is unclear in the post-intervention design what methodology or strategy was implemented to address the nomadic population described in the context.

Author response: We thank the reviewer for highlighting the need for clarity regarding the methodology or strategy implemented to address the nomadic population in the post-intervention phase. As part of our community participatory approach, our **"Effective Communication and Community Engagement" strategy** was specifically designed to include approaches tailored to reach this population. This strategy incorporated **engagement of nomadic community leaders** and **information dissemination through their existing community networks**, as well as **flexible timing** of MDA delivery to accommodate their movements. These components are elaborated in detail within the **"The tested interventions"** section of our methodology found in lines 263 - 291 in the revised manuscript.

**Discussion**

21. Table 3 suggests that practicing a religion other than Christianity or not practicing any religion is a protective factor or predictor of better MDA coverage, compared to Christianity. Is there any hypothesis regarding this result? It is considered appropriate to mention this in the discussion.

Author response: Thank you for pointing out the significant finding in Table 4 (formerly Table 3) regarding the association between religious affiliation and better MDA coverage in the post-intervention phase. While this was an interesting finding in the post-intervention data, it did not show a significant impact on MDA access when we assessed the overall effect of our intervention. As our primary focus was on identifying factors that demonstrated a significant impact across the pre- and post-intervention periods, we concentrated our discussion on those key variables. However, we acknowledge the potential for this observation, and this can be explored in future research.

21. The frequency of the adverse effects described in Lines 342 to 345 is quite significant. It is considered appropriate to mention these values in comparison to those reported by other researchers, to understand whether this is something that is negatively impacting the participation of people in that district in MDA rounds.

Author response: Thank you for raising this point about the side effects. It is important to clarify that the "adverse effects" data we collected in this section of the survey reflects the **information participants recalled being provided by drug distributors regarding treatment *potential* side effects**, rather than directly reported experienced adverse events. We have revised this paragraph appropriately, see lines 799-802.

Reviewer #2: Thank you for allowing me to review this manuscript. The study is important for evaluating access to MDA in a particularly challenging region of Kenya. As the trachoma program nears its endgame, these areas will only increase in relevance to the global program, so an understanding of these areas is key! This work is significant for that purpose.

>> Author response: Thanks for your positive comment.

The strengths and weaknesses are well-noted within the manuscript. In my opinion, the analysis of factors associated with MDA access pre- and post-intervention is clear. However, the analysis of the tested interventions is less so. As the authors mention, the lack of a control group here is problematic for drawing conclusions, so I'd advise the authors to be careful in their interpretation of these data.
>> Author response: We appreciate the reviewer's observation regarding the clarity of the analysis of factors associated with MDA access compared to the analysis of the tested interventions. We fully agree that the lack of a control group presents a significant limitation in definitively attributing the observed changes in MDA access solely to our interventions. We have discussed and interpreted the results cautiously within the limits of the study design. We have carefully framed our conclusions, acknowledging that without a control group, we cannot definitively rule out the influence of other factors or secular trends.

In addition, since there's no specific space to input comments on the abstract and introduction, please see below:

In the abstract, please rephrase line 72 to avoid the distinction of being a housewife as not being a "meaningful job". (Perhaps it may be more correct to say a job outside the home or a wage-earning job?)
>> Author response: We thank the reviewer for raising this important point in the abstract. We agree that the original wording may unintentionally devalue the significant contributions of housewives. We have revised the abstract to use more precise and neutral language. For example, we have rephrased the sentence to refer to 'individuals not engaged in work outside the home' instead of the original phrasing. Please see line 77.

In the introduction, line 90, please edit to reflect that trachoma is the leading infectious cause of blindness (not leading cause of blindness generally).

>> Author response: Thank you for this point. We have edited the specific line to clarify that trachoma is the leading *infectious* cause of blindness, see line 111.

In line 99, is the recommendation based on 10% in the whole population, or children 1-9? Please provide a citation here.

>> Author response: We have added the requested citation to line 121 to support the recommendation. The recommendation is mass treatment for a minimum period of 3 years where the prevalence is 10% or more in children 1-9 years.
