# Supplementary material for: Assessing the effectiveness of the community participation approaches to improve access to mass drug administration for trachoma elimination in a pastoral conflict area of Baringo County, Kenya
Source: PLoS Negl Trop Dis. 2025 Aug 11;19(8):e0013408. doi: 10.1371/journal.pntd.0013408 (PMC12360648; doi:10.1371/journal.pntd.0013408)
Supplement: Attachment — Submitted filename: Response to reviewers.docx [file pntd.0013408.s005.docx]

Reviewer #1: The objective of the study was “to evaluate the effectiveness of a community-based participatory approach implemented to improve the adoption of MDA for trachoma control”; However, analyses are performed and conclusions are drawn only from univariate/bivariate analyses, meaning the model is not adjusted for potential confounding factors; therefore, the ORs may be under- or overestimated. Therefore, the main recommendation is to perform multivariate analyses to adjust the ORs and their confidence intervals, or, in another scenario, to declare the limitation in the interpretation of the associations presented, given that confounding factors were not controlled.

Author response: We appreciate your insightful comment regarding the need for multivariable analysis to account for potential confounding factors. We have now performed multivariable analysis and revised the results section appropriately. Please see Tables 5 and 8 in the revised manuscript.

Reviewer #2: My comments from the previous draft have been appropriately addressed. Many thanks to the authors for this additional effort.

**Results**

Reviewer #1: The results are presented clearly, however, in line with the above, a multivariate analysis is needed.

Author response: We appreciate your insightful comment regarding the need for multivariable analysis to account for potential confounding factors. We have now performed multivariable analysis and revised the results section appropriately. Please see Tables 5 and 8 in the revised manuscript.

Reviewer #2: My comments from the previous draft have been appropriately addressed. Many thanks to the authors for this additional effort. Some of the text in the figures is a bit hard to read; however this is likely an issue of the files being compressed as part of the submission system. Please verify the text in the actual figure files is sufficiently clear.

Author response: Thanks for your comments on the figure quality. We have confirmed, through the PLOS PACE system, that the figures are clear.

**Conclusions**

Reviewer #1: The results present high ORs with very wide confidence intervals; although the OR is high (20.12) and the association is statistically significant (p = 0.007), the fact that the CI ranges from 2.29 to 176.96 suggests that the precision of the estimate is low.
This does not invalidate the association, but it does indicate that the result should be interpreted with caution. This limitation should be mentioned in the discussion of the article. Thank you

Thank you for this comment. This is well noted and agreeable that some estimates have low precision. We have included this as a caution to be taken when interpreting the results. Please see lines 841-844.

Reviewer #2: My comments from the previous draft have been appropriately addressed. Many thanks to the authors for this additional effort.

**Editorial and Data Presentation Modifications?**

Reviewer #1: Lines 96- 100: The threshold defined by the WHO for mass antibiotic administration is ≥5%, not 10% as stated in the text.

This observation persists in the manuscript; it is widely acknowledged that districts with TF prevalences of 5% to 9.9% should also conduct a round of MDA. A reference is attached. In this same paragraph it is not clear what prevalence is, that is, it is not clear what prevalence of TF is. Reference:
[https://pmc.ncbi.nlm.nih.gov/articles/PMC3681669/#:~:text=Background,or%20discontinue%20MDA%2C%20are%20unknown.](https://pmc.ncbi.nlm.nih.gov/articles/PMC3681669/%23:~:text=Background,or%20discontinue%20MDA%2C%20are%20unknown.)

Author response: Thanks for your comment on the MDA threshold. Our statement is clear that MDA should be implemented in the whole population in communities where the prevalence is more than 10% in children aged 1 to 9 years. We used guidance provided on “*Report of the 3^rd^ Global Scientific Meeting on Trachoma*” pages 6 and 10. See the link for the reference: https://iris.who.int/bitstream/handle/10665/329074/WHO-PBD-2.10-eng.pdf?sequence=1&isAllowed=y

Lines 202-206 . Please check: “Although the treatment coverage achieved in 2020 and 2021 was 95.6% and 79.7%, 203 respectively, further investigation at the lower levels (wards) showed coverages ranging 204 between 48% to 57%, which are far below the recommended threshold. Investigations in 205 Loyamorok ward showed low prevalence, ranging from 56.6% and 67.6% respectively in 2020 206 and 2021”.

Specifically on line 205 I think it is necessary to adjust by referring to Coverage and not to prevalence”

Author response: We appreciate the reviewer for this observation. We’ve adjusted by referring to treatment coverage and not prevalence.

Reviewer #2: My comments from the previous draft have been appropriately addressed. Many thanks to the authors for this additional effort.

**Summary and General Comments**


Reviewer #1: Thank you again for the opportunity to review this manuscript. Overall, I believe most of the comments were incorporated or taken into account in the new version, which I consider to be clearer.

My main recommendation is to perform a multivariate analysis to control for confounding factors and avoid over- or underestimation of the ORs used; this would undoubtedly improve the quality of the results and the study in general.

Author response: We appreciate your insightful comment regarding the need for multivariable analysis to account for potential confounding factors. We have now performed multivariable analysis and revised the results section appropriately. Please see Tables 5 and 8 in the revised manuscript.

Reviewer #2: My comments from the previous draft have been appropriately addressed. Many thanks to the authors for this additional effort.
